# X-ray structure of the direct electron transfer-type FAD glucose dehydrogenase catalytic subunit complexed with a hitchhiker protein

**DOI:** 10.1107/S2059798319010878

**Published:** 2019-08-28

**Authors:** Hiromi Yoshida, Katsuhiro Kojima, Masaki Shiota, Keiichi Yoshimatsu, Tomohiko Yamazaki, Stefano Ferri, Wakako Tsugawa, Shigehiro Kamitori, Koji Sode

**Affiliations:** a Life Science Research Center and Faculty of Medicine, Kagawa University, 1750-1 Ikenobe, Miki-cho, Kita-gun, Kagawa 761-0793, Japan; b Department of Biotechnology and Life Science, Graduate School of Engineering, Tokyo University of Agriculture and Technology, 2-24-16 Naka-cho, Koganei, Tokyo 184-8588, Japan; c Department of Chemistry, Missouri State University, Springfield, MO 65897, USA; d Research Center for Functional Materials, National Institute for Materials Science (NIMS), 1-2-1 Sengen, Tsukuba, Ibaraki 305-0047, Japan; e Department of Applied Chemistry and Biochemical Engineering, Shizuoka University, 3-5-1 Johoku, Naka-ku, Hamamatsu, Shizuoka 432-8561, Japan; f Joint Department of Biomedical Engineering, University of North Carolina at Chapel Hill and North Carolina State University, Chapel Hill, NC 27599, USA

**Keywords:** direct electron transfer, flavin adenine dinucleotide-dependent dehydrogenase complex, glucose dehydrogenase, iron–sulfur cluster, hitchhiker protein, glucose sensors, *Burkholderia cepacia*

## Abstract

The bacterial flavin adenine dinucleotide (FAD)-dependent glucose dehydrogenase complex derived from *Burkholderia cepacia* (BcGDH) is a representative molecule of direct electron transfer-type FAD-dependent dehydrogenase complexes. In this study, the X-ray structure of BcGDHγα, the catalytic subunit (α-subunit) of BcGDH complexed with a hitchhiker protein (γ-subunit), was determined. The most prominent feature of this enzyme is the presence of the 3Fe–4S cluster, which is located at the surface of the catalytic subunit and functions in intramolecular and intermolecular electron transfer from FAD to the electron-transfer subunit. The structure of the complex revealed that these two molecules are connected through disulfide bonds and hydrophobic interactions, and that the formation of disulfide bonds is required to stabilize the catalytic subunit. The structure of the complex revealed the putative position of the electron-transfer subunit. A comparison of the structures of BcGDHγα and membrane-bound fumarate reductases suggested that the whole BcGDH complex, which also includes the membrane-bound β-subunit containing three heme *c* moieties, may form a similar overall structure to fumarate reductases, thus accomplishing effective electron transfer.

## Abstract 2

The X-ray structure of the catalytic subunit of *Burkholderia cepacia* FAD glucose dehydrogenase complexed with a hitchhiker protein was determined as a representative molecule of direct electron transfer-type FAD-dependent dehydrogenase complexes. The 3Fe–4S cluster is present at the surface of the catalytic subunit and serves in the intramolecular and intermolecular electron transfer from FAD to the electron-transfer subunit.

## 1. Introduction   

Various sugar oxidoreductases (dehydrogenases) have been reported to be inherently capable of direct electron transfer to electrodes composed of carbon materials or to gold electrodes. These dehydrogenases harbor an electron-transfer domain or subunit, together with a catalytic domain or subunit. The catalytic domains or subunits, which are responsible for catalyzing sugar oxidation, are categorized by their cofactor: flavin adenine dinucleotide (FAD) or pyrroloquinoline quinone (PQQ). The electron-transfer domains or subunits, which are responsible for transferring electrons to the external electron acceptor, are also categorized by the type of heme (heme *b* or *c*) that is present in the electron-transfer domain or subunit.

One of the representative groups of direct electron transfer-type (DET-type) dehydrogenases consists of cellobiose dehydrogenases (CDHs), which are composed of a catalytic domain harboring FAD and a heme *b*-type electron-transfer domain. In CDHs, open and closed states were identified by the approach of the cytochrome domain to the catalytic domain via a flexible linker (Tan *et al.*, 2015 ▸).

The other representative protein group that is capable of direct electron transfer consists of FAD-dependent dehydro­genase complexes. These complexes are composed of a catalytic subunit with FAD, an electron-transfer subunit containing three heme *c* moieties and a small subunit. The isolation, characterization, bioelectrochemical studies and application of several bacterial FAD-dependent dehydro­genase complexes have been reported, including bacterial glucose dehydrogenase (FADGDH; Inose *et al.*, 2003 ▸; Sode *et al.*, 1996 ▸; Tsuya *et al.*, 2006 ▸; Yamazaki *et al.*, 1999 ▸; Yamaoka & Sode, 2007 ▸; Yamaoka *et al.*, 2008 ▸), fructose dehydrogenase (FDH; Ameyama *et al.*, 1981 ▸; Kawai *et al.*, 2013 ▸;), 2-keto-d-gluconate dehydrogenase (KGDH; Kataoka *et al.*, 2015 ▸; Shinagawa *et al.*, 1981 ▸) and sorbitol dehydrogenase (Toyama *et al.*, 2005 ▸). These FAD-dependent dehydrogenase complexes have the potential to directly transfer electrons to an electrode because of the presence of the heme *c* subunit. However, no structural information is currently available for any subunits from DET-type FAD-dependent dehydrogenase complexes.

Our research group has been studying a representative FAD-dependent dehydrogenase complex (FADGDH) derived from *Burkholderia cepacia* SM4 (BcGDH). BcGDH comprises three distinct subunits: the catalytic subunit (α-subunit), which contains an FAD cofactor in its redox center, shows catalytic activity and oxidizes the first hydroxyl group of glucose, the small subunit (γ-subunit), a hitchhiker protein of the bacterial TAT secretion system that is necessary for the proper folding and secretion of the α-subunit (Yamaoka *et al.*, 2004 ▸), and the membrane-bound subunit with three heme *c* moieties (β-subunit) that is responsible for the transfer of electrons between the active-site cofactor and external electron acceptors. Owing to the presence of the β-subunit, BcGDH is capable of transferring electrons directly to an electrode, making it an ideal molecule for glucose sensors and applications in a variety of biomedical devices (Sode *et al.*, 2016 ▸; Yamashita *et al.*, 2018 ▸). In addition, the BcGDHγα complex, which is BcGDH lacking the β-subunit, also exhibits dye-mediated glucose dehydrogenase activity (Inose *et al.*, 2003 ▸). Recently, based on biochemical analyses and electron paramagnetic resonance spectroscopy, we reported the presence of a 3Fe–4S cluster in the catalytic subunit (Shiota *et al.*, 2016 ▸). The 3Fe–4S cluster is located in the cysteine-rich region, which is conserved in the catalytic subunits of previously reported FAD-dependent dehydro­genase complexes. The 3Fe–4S cluster is responsible for electron transfer from FAD (intramolecular) to the multiheme *c* subunit (intermolecular), which is the key position for understanding the features of this group of enzymes that are capable of direct electron transfer.

Another notable feature of the enzymes in the FAD-dependent dehydrogenase complex is the presence of a small subunit that is essential for the functional expression of the FAD-harboring catalytic subunit. Considering their primary structure, particularly the signal sequences necessary for secretion, these small subunits are predicted to be hitchhiker proteins that are needed for secretion of the catalytic subunit into the periplasmic space (Yamaoka *et al.*, 2004 ▸). However, no structural information is available for any types of hitchhiker proteins or their complexes with targeted proteins.

In this study, we determined the X-ray structure of BcGDHγα, consisting of the BcGDH catalytic subunit complexed with the small (hitchhiker) subunit. The structure of the FAD-binding catalytic subunit was similar to those of several glucose-methanol-choline (GMC) oxidoreductases. The catalytic sites of BcGDHγα were conserved compared with other GMC oxidoreductases. In the structure of BcGDHγα, the 3Fe–4S cluster was located at the surface of the catalytic subunit. The structure of the complex of the catalytic subunit with the small subunit revealed that these two molecules were connected through disulfide bonds and hydrophobic interactions. Site-directed mutagenesis studies were performed to elucidate the role of the disulfide bond. The structural similarities to other FAD-dependent dehydro­genase complexes and to fumarate reductase are also discussed.

## 2. Materials and methods   

### 2.1. Recombinant expression of BcGDHγα   

The structural genes encoding the catalytic (α) and small (γ) GDH subunits of *B. cepacia* sp. SM4 (FERMBP-7306) were subcloned into the high-expression vector pTrc99A with a His tag at the C-terminus of the α-subunit. The constructed plasmid (designated pTrcγα-His) was transformed into the bacterial host *Escherichia coli* BL21 (DE3) for expression. In the present study, a complex of the γ-subunit and His-tagged α subunit was expressed as recombinant wild-type BcGDHγα. The γ-subunit consists of 168 amino acids (18 kDa), including 47 amino acids of the signal peptide at the N-terminal region, and the mature γ-subunit contains 121 amino acids (13 kDa). The α subunit consists of 539 amino acids and was produced as a catalytic domain of 60 kDa.

Transformed *E. coli* were cultured in 500 ml conical flasks containing 100 ml ZYP-5052 medium (Studier, 2005 ▸) in a rotary shaker at 293 K for 48 h.

The *E. coli* selenium auxotroph strain B834 (DE3) was used to produce selenomethionine-containing BcGDHγα. The *E. coli* B834 (DE3) cells harboring pTrcγα-His were cultured in 500 ml conical flasks containing 100 ml PASM-5052 medium (Studier, 2005 ▸) in a rotary shaker at 293 K for 211 h.

The cells were harvested by centrifugation and then resuspended in 20 m*M* sodium phosphate buffer containing 20 m*M* imidazole and 0.5 *M* NaCl pH 7.0. After resuspension, the cells were disrupted with a French press. The lysate was centrifuged at 10 000*g* for 15 min at 277 K to remove the the insoluble fraction consisting of cell debris and inclusion bodies. The resulting supernatant, which was designated the crude extract, was purified by FPLC.

### 2.2. Enzyme purification   

The recombinant BcGDHγα complex was purified using nickel-chelate chromatography and cation-exchange chromatography. The crude extract was loaded onto a HisTrap HP column (1 ml; GE Healthcare Life Sciences, Uppsala, Sweden) that had been equilibrated with 20 m*M* sodium phosphate buffer containing 20 m*M* imidazole and 0.5 *M* NaCl pH 7.0 and was washed with the same buffer. GDH was then eluted with ten column volumes of a stepwise imidazole gradient (70, 380 and 500 m*M* imidazole in 20 m*M* sodium phosphate buffer and 0.5 *M* NaCl pH 7.0) at a rate of 1 ml min^−1^ for each step. The fractions with the highest activities were pooled and dialyzed overnight in 10 m*M* potassium phosphate buffer pH 6.0.

The pooled fractions were subsequently loaded onto a Resource S column (5 ml; GE Healthcare, Little Chalfont, England) that had been equilibrated with 10 m*M* potassium phosphate buffer pH 6.0 and were washed with the same buffer. GDH was eluted with 20 column volumes of a linear NaCl gradient (0–1 *M* NaCl in 10 m*M* potassium phosphate buffer pH 6.0) at a rate of 5 ml min^−1^. The purified enzyme was concentrated to 8.9 mg ml^−1^ and the buffer was exchanged to Milli-Q water using Amicon Ultra-15 (nominal molecular-weight limit 3000; Merck Millipore, Carrigtwohill, Ireland). The protein concentrations were measured using a DC Protein Assay kit (Bio-Rad Laboratories, Hercules, California, USA).

### 2.3. Site-directed mutagenesis   

Site-directed mutagenesis of the target amino acids (Cys213 in the α-subunit and Cys152 in the γ-subunit) was accomplished using the QuikChange mutagenesis kit (Agilent, Santa Clara, California, USA) according to the manufacturer’s instructions. All mutations were confirmed by nucleotide sequencing.

### 2.4. Enzyme assay   

The activities of crude extracts and the purified recombinant BcGDHγα complex were determined using methods described in a previous study (Inose *et al.*, 2003 ▸) with slight modifications. The enzyme sample was incubated at room temperature with 10 m*M* potassium phosphate buffer pH 7.0 containing 6 m*M* 5-methylphenazinium methylsulfate (phenazine methosulfate; PMS), 0.06 m*M* 2,6-dichlorophenolindo­phenol (DCIP) and various concentrations of glucose. The activity was determined by monitoring the decrease in the absorbance of DCIP at 600 nm and using the molar absorption coefficient of DCIP (16.3 m*M* cm^−1^ at pH 7.0) to calculate the enzyme activity. The molar absorption coefficient of DCIP was determined by measuring the absorbance of fixed concentrations of DCIP at 600 nm in 10 m*M* potassium phosphate buffer pH 7.0. One unit of enzyme activity is defined as the amount of enzyme that oxidizes 1 µmol glucose per minute.

### 2.5. Crystallization   

Initial crystal screening was performed using the sitting-drop vapor-diffusion method with a Mosquito system (TTP Labtech, Hertfordshire, England). The protein concentration of wild-type BcGDHγα was 8.9 mg ml^−1^ in Milli-Q water. After a few days, yellow crystals were observed in a reservoir solution containing 60% Tacsimate pH 7.0. Well diffracting crystals were obtained in a droplet containing a mixture of 1.5 µl protein solution (5.7 mg ml^−1^ in Milli-Q water) and 0.75 µl reservoir solution (59.9–60.2% Tacsimate pH 7.0) in a well containing 50 µl reservoir solution using the sitting-drop method at 293 K.

The methionines in BcGDHγα were replaced with selenomethionines (SeMet BcGDHγα) in order to determine the initial phases for the structure factors of wild-type BcGDHγα. However, crystals of SeMet BcGDHγα were not obtained under the same conditions as those of wild-type BcGDHγα. Since the prepared SeMet BcGDHγα contained a small amount of nonprocessed γ-subunit (18 kDa), which was observed on SDS–PAGE gels, the crystallization of SeMet BcGDHγα was attempted in the presence of proteases using Proti-Ace (Hampton Research, California, USA). Some crystals of SeMet BcGDHγα appeared in a droplet consisting of 1.0 µl protein solution (10.2 mg ml^−1^ in Milli-Q water), 0.2 µl of a 0.1 mg ml^−1^ subtilisin solution and 1.0 µl reservoir solution (60% Tacsimate pH 7.0) in a well containing 50 µl reservoir solution using the sitting-drop method at 293 K.

### 2.6. X-ray crystallography   

Single crystals of both wild-type BcGDHγα and SeMet BcGDHγα were mounted in cryoloops and directly flash-cooled in a stream of nitrogen gas at 100 K. X-ray diffraction data were collected using an ADSC Quantum 270 CCD detector system on the PF-AR NE3A beamline at the High Energy Accelerator Research Organization (KEK), Tsukuba, Japan. Diffraction data were processed using *HKL*-2000 (Otwinowski & Minor, 1997 ▸) and the *CCP*4 suite (Winn *et al.*, 2011 ▸). Although X-ray diffraction data were collected from the crystal of BcGDHγα to 2.2 Å resolution, the data actually used for structure determination were truncated at 2.6 Å resolution owing to an extensively high *R*_merge_ in the outermost shell. The unit-cell parameters of the crystal were large (*a* = *b* = 110.5, *c* = 524.9 Å) and the diffraction pattern was characterized by strong anisotropy.

The initial phases of SeMet BcGDHγα were obtained using the single-wavelength anomalous dispersion (SAD) method with the *AutoSol* program (Terwilliger, 2004 ▸). Since the unit-cell parameters of the crystal of wild-type BcGDHγα were isomorphous to those of the SeMet derivative, the phases were transferred directly to the former and the model was constructed using *AutoBuild* in the *PHENIX* system (Adams *et al.*, 2010 ▸; Afonine *et al.*, 2012 ▸).

Further model building and structure refinement were performed using *Coot* (Emsley *et al.*, 2010 ▸) and *REFMAC*5 (Murshudov *et al.*, 2011 ▸), respectively. The structure was validated using *PROCHECK* (Laskowski *et al.*, 1993 ▸, 2001 ▸).

The anomalous dispersion of Fe atoms was utilized in order to determine the number and the positions of Fe atoms in the iron–sulfur cluster. A SAD data set from a crystal of wild-type BcGDHγα was collected to 1.74086 Å resolution on beamline PF-AR NW12A at KEK using an ADSC Quantum 210r CCD detector system.

Data-collection and refinement statistics for all data sets are listed in Table 1 ▸. Figs. 1, 2, 3, 4, 6 and Supplementary Figs. S2, S5, S6, S7 and S9 were generated using *PyMOL* (Schrödinger, New York, USA).

## 3. Results   

### 3.1. The overall structure of BcGDHγα   

**Figure 1:**
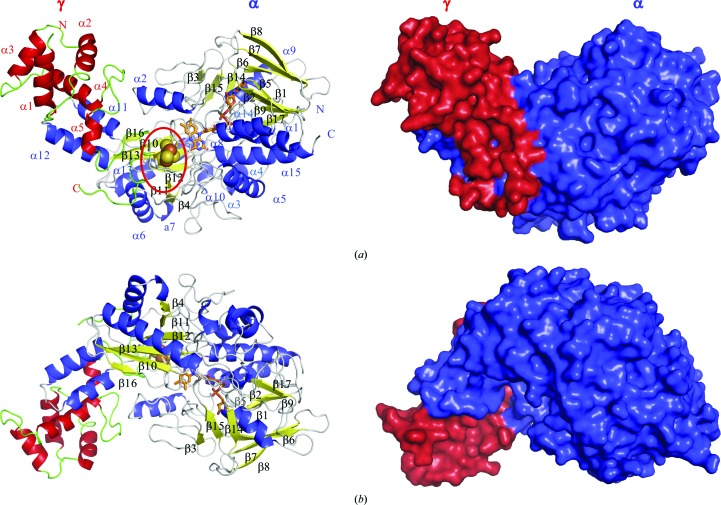
Overall structure of BcGDHγα. (*a*) Overall structure of BcGDHγα shown as cartoon (left) and surface (right) models. In the cartoon model, the γ-­subunit is shown as red α-helices and green loop regions, and the α-subunit is represented with blue α-helices, yellow β-strands and gray loop regions. In the surface model, the γ- and α-subunits are shown in red and blue, respectively, and are tightly bound to each other. The bound FAD is represented as an orange stick model. The cysteine cluster indicated with a red circle includes the iron–sulfur cluster shown as spheres. (*b*) A view of the model rotated 180° around the *x* axis.

Fig. 1 ▸ and Supplementary Fig. S1 show the overall structure of the complex of the γ- and α-subunits of BcGDH and the topology of the protein, respectively. The γ-subunit consists of five α-helices (colored red). The overall structure of the α-subunit comprises 15 α-helices (colored blue) and 17 β-strands and adopts an FAD-binding fold. The additional domain contains a six-stranded antiparallel β-sheet surrounded by six α-helices and a protruding loop including two α-helices (α11 and α12) facing towards the γ-subunit. Two distinguishing long loop regions are located between β2 and β3 and between β4 and α8. The former loop (Ala39–Leu83) contains a unique α-helix (α2) that is located in close prox­imity to the γ-subunit at the center of the γα-subunit complex and above the iron–sulfur cluster shown in the red circle [Fig. 1 ▸(*a*)], which is described later. The latter loop (Glu197–Asn229) surrounds the iron–sulfur cluster and contains a cysteine cluster (Cys212, Cys213, Cys218 and Cys222); three of the four cysteines are involved in forming the 3Fe–4S cluster.

**Figure 2:**
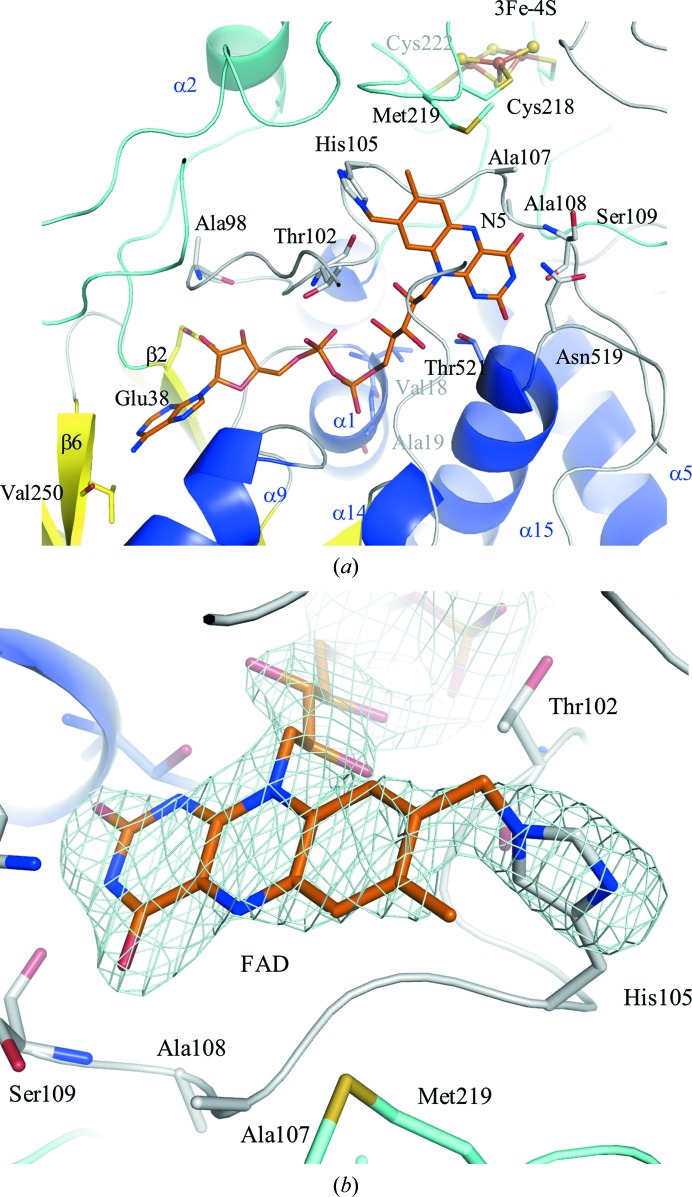
FAD-binding site and FAD covalently bound to His105 of the α-subunit. (*a*) FAD-binding site with the surrounding environment. A long loop of α-­subunit including α2 is colored cyan. FAD is shown as an orange stick model. Other colors are the same as in Fig. 1 ▸. (*b*) The simulated-annealing OMIT maps of FAD and His105 in wild-type GDHγα contoured at 4σ are shown in light blue. FAD is shown as an orange stick model.

The structure around FAD in the α-subunit is shown in Fig. 2 ▸. FAD is surrounded by part of the long loop between Ala98 and Ser109, α1, α15, β2 and β6. Since continuous electron density was observed between FAD and His105 in the α-subunit of BcGDHγα [Fig. 2 ▸(*b*)], a covalent bond was deemed to form between FAD and His105 in the α-subunit of BcGDH.

In the crystal, two pairs of BcGDHγα complexes were observed in the asymmetric unit (Supplementary Fig. S2). As shown in the surface model presented in Figs. 1 ▸(*a*) and 1 ▸(*b*), BcGDHγα forms a heterodimer with a bent form, and the bent complex (γα-subunits) faces the back side of the other complex in the crystal (Supplementary Fig. S2). Although the protein seems to form a heterotetramer, γαγ′α′, according to *PISA* analysis (http://www.ebi.ac.uk/pdbe/pisa/) this assembly is unstable and forms two pairs of γ- and α-subunits (γα and γ′α′). A gel-filtration chromatogram revealed the absence of an oligomeric form of BcGDHγα in solution (data not shown). Therefore, the observed γαγ′α′ heterotetramer in the crystal structure is an artifact of crystallization. Since each molecule of γα and γ′α′ is almost identical, with r.m.s. deviations for C^α^ atoms of 0.48 (α and α′) and 0.89 (γ and γ′), the structural description concentrates on γα unless otherwise specified.

### 3.2. Iron–sulfur cluster   

**Figure 3:**
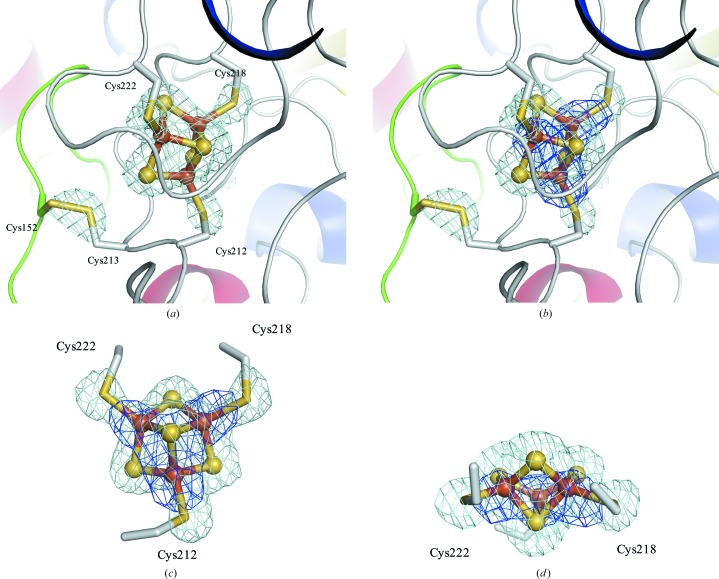
Cysteine cluster of BcGDHγα. A unique cysteine cluster is formed by one cysteine from the γ-subunit and four cysteines from the α-subunit. Cys152 and Cys213 form a disulfide bond between the γ- and α-subunits. Cys212, Cys218 and Cys222 are involved in the iron–sulfur cluster (3Fe–4S). The identified positions of the 3Fe–4S cluster and the disulfide bond between the γ- and α-subunits are shown. Simulated-annealing OMIT maps of F3S (the 3Fe–4S iron–sulfur cluster) and five cysteine residues (Cys152, Cys212, Cys213, Cys218 and Cys222) in wild-type BcGDHγα contoured at 5σ are shown in light blue in (*a*)–(*d*). The 2*F*_o_ − *F*_c_ electron-density maps using the merged data sets of wild-type BcGDHγα collected at wavelengths of 1.0 and 1.74086 Å (after the initial refinement and before including F3S in refinement) are shown in blue and contoured at 5σ in (*b*), (*c*) and (*d*). S and Fe atoms are shown in yellow and orange, respectively. The identified 3Fe–4S cluster consists of three Fe atoms and four S atoms represented as spheres.

The locations of Fe atoms were identified using the anomalous dispersion method and were observed in the expected positions of the iron–sulfur cluster. Three Fe-atom sites were identified in the iron–sulfur cluster of each molecule [Figs. 3 ▸(*a*)–3 ▸(*d*)]. The simulated-annealing OMIT maps of sulfur ions in the iron–sulfur cluster and the disulfide bond indicated that this iron–sulfur cluster is a 3Fe–4S cluster coordinated by Cys212, Cys218 and Cys222 of the α-subunit. A neighboring cysteine, Cys213, in the α-subunit forms a disulfide bond with Cys152 of the γ-subunit. The unique cysteine cluster of BcGDHγα contributes to the formation of the 3Fe–4S cluster for electron transfer and the disulfide bond for stabilization of the structure of the γα complex.

### 3.3. Interface between the γ- and α-subunits   

**Figure 4:**
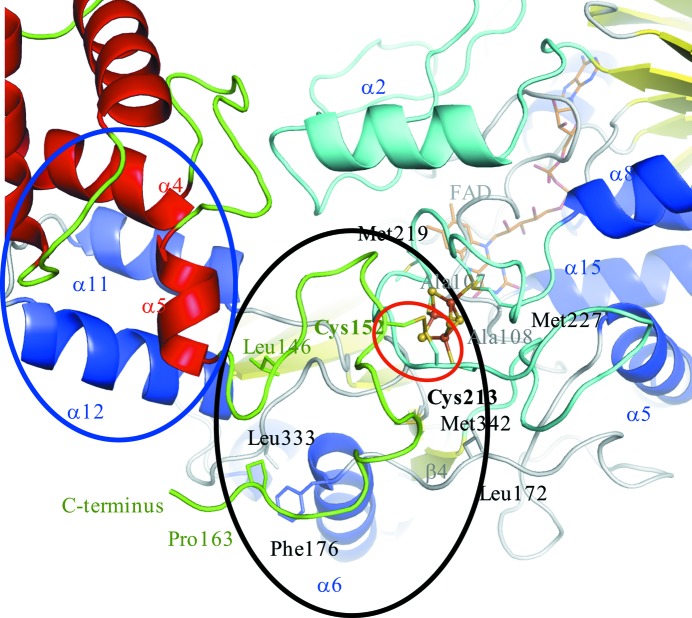
Tightly bound interface between the γ- and α-subunits. The highly hydrophobic C-terminal tail of the γ-subunit (colored green) including Cys152 of the γ-subunit covers the contact areas like a lid at the interface. The disulfide bond between subunits is indicated by a red circle. One of the long loops of the α-subunit including α2 is colored cyan and contacts the C-terminal loop region of the γ-subunit. The other colors are the same as in Fig. 2 ▸. α4 and α5 of the γ-subunit also make contacts with protruding helices (α11 and α12) of the α-subunit in the area indicated with a blue circle.

The γ-subunit tightly binds the α-subunit and forms a stable heterodimer. The hydrophobic loop region at the C-terminus of the γ-subunit (colored green in the black circle in Fig. 4 ▸) contacts one of the distinguishing long loops, including α2 in the α-subunit at the interface (colored in cyan). A disulfide bond is located at the interface between the γ- and α-subunits (Cys152 in the γ-subunt and Cys213 in the α-subunit), as indicated by the red circle in Fig. 4 ▸, and is in close proximity to the hydrophobic cluster created by the C-terminal region in the γ-subunit indicated by the black circle (Leu146, Val147, Ile148, Pro153, Pro156, Gly157, Phe158, Trp159, Ala160 and Pro163) and the surrounding residues of the α-subunit (Leu172, Pro173, Leu174, Phe176, Leu333, Trp334, Pro335, Gly336, Gly338, Pro339 and Met342). A 3Fe–4S cluster is located next to the disulfide bond, and the iron–sulfur cluster is located in the hydrophobic environment formed by the above hydrophobic cluster, the hydrophobic loop region (Met219, Pro223, Ile224, Ala226 and Met227, colored cyan) of the α-subunit, including four cysteine residues (Cys212, Cys213, Cys218 and Cys222) involved in formation of the 3Fe–4S cluster and the disulfide bond, and two alanine residues (Ala107 and Ala108) located between 3Fe–4S and the iso­alloxazine ring of FAD. Furthermore, α4 and α5 of the γ-subunit make contacts with protruding helices, including the α11 and α12 helices of the α-subunit (in the area indicated by the blue circle). These hydrophobic contacts contribute to the tightly bound subunit interface.

### 3.4. Site-directed mutagenesis   

**Figure 5:**
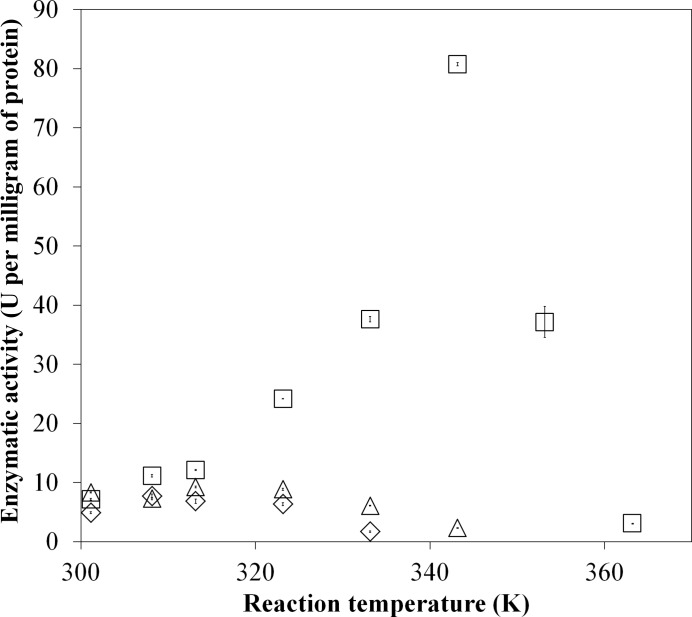
The effect of temperature on the enzymatic activities of wild-type and mutant BcGDHγα. Dye-mediated glucose dehydrogenase activities of wild-type (open squares), γ(Cys152Ser)α (open triangles) and γα(Cys213Ser) (open diamonds) BcGDH at each temperature are shown.

The crystal structure of the BcGDHγα complex revealed the presence of an inter-subunit disulfide bond between the side chains of Cys213 in the α-subunit and Cys152 in the γ-subunit. As reported previously (Shiota *et al.*, 2016 ▸), substitution of Cys213 in the α-subunit by serine [γα(Cys213Ser)] only exerts a limited effect on the kinetic parameters of the BcGDHγα complex at room temperature. Thus, the inter-subunit disulfide bond is not essential for enzyme activity. A mutant BcGDHγα complex with a Cys152Ser mutation in the γ-subunit [γ(Cys152Ser)α] was constructed and characterized to further confirm this hypothesis. Table 2 ▸ presents the kinetic parameters of the mutant BcGDHγα complex at room temperature. In the absence of the electron-transfer subunit (β-subunit), the BcGDHγα complex shows relatively low dye-mediated glucose dehydrogenase activity at the conventionally used concentration of the primary electron accepter (PMS; Yamazaki *et al.*, 1999 ▸). Therefore, the enzyme activity of the BcGDHγα complex was determined using 6 m*M* PMS, a concentration that is tenfold higher than the condition used for the BcGDH complex containing the electron-transfer subunit (Supplementary Fig. S4). The *V*_max_ of the γ(Cys152Ser)α complex is only moderately lower than that of the wild-type γα complex. The *K*_m_ of the γ(Cys152Ser)α complex is comparable to the *K*_m_ of the wild-type γα complex. While the substitutions of these cysteine residues did not substantially affect the kinetic parameters of the BcGDHγα complex at room temperature, disulfide bonds often contribute to the stability of the tertiary and/or quaternary structure of proteins. We therefore studied the enzyme activity of the cysteine-substituted mutants at higher temperatures. The wild-type BcGDHγα complex showed maximum activity at 343 K owing to its high thermal stability, whereas both the γ(Cys152Ser)α and γα(Cys213Ser) complexes showed maximum activity at approximately 303–313 K (Fig. 5 ▸). A substantial decrease in the optimal reaction temperatures of the γ(Cys152Ser)α and γα(Cys213Ser) complexes suggested an important role of the inter-subunit disulfide bond in maintaining the thermal stability of the wild-type BcGDHγα complex.

## 4. Discussion   

A *DALI* search (Holm & Rosenström, 2010 ▸; Holm & Laakso, 2016 ▸) revealed many proteins that are structurally similar to the α-subunit of BcGDHγα, with high *Z*-scores in the range 23.4–33.0 (Supplementary Table S1). These enzymes are categorized as members of the FAD-containing glucose-methanol-choline oxidoreductase (GMC) family.

Among the identified enzymes in the GMC family, pyranose 2-oxidase (P2Ox) shows the highest similarity to the BcGDH α-subunit [Supplementary Figs. S5(*b*) and S5(*d*)]. The structure of cholesterol oxidase (ChOx) is also similar to the structure of the BcGDH α-subunit [Supplementary Figs. S5(*c*) and S5(*e*)].

The residues responsible for the catalytic reaction have previously been identified in a variety of GMC oxido­reductases. A His/His pair in GOx, fungal FADGDH, pyran­ose dehydrogenase (PDH) and aryl-alcohol oxidase (AAOx), and a His/Asn pair in cholesterol oxidase (ChOx), cellobiose dehydrogenase (CDH), choline oxidase (COx) and P2Ox are expected to function as catalytic pairs based on the crystal structures, site-directed mutagenesis, pH-dependence studies or theoretical calculations. The catalytic pair in BcGDH is likely to be His/Asn, represented by His476 and Asn519, and corresponds to His689 and Asn732 in cellobiose dehydro­genase.

The structure of the active site of BcGDHγα was compared with *Phanerochaete chrysosporium* cellobiose dehydrogenase (PcCDH) bound to 6-hydroxy-FAD and the inhibitor cellobionolactam (ABL; Hallberg *et al.*, 2003 ▸; Supplementary Fig. S6). The His/Asn catalytic pairs (His476/Asn519 in BcGDHγα and His689/Asn732 in PcCDH) and the residues recognizing the position of the glucose moiety at the nonreducing end are conserved. Asn688 of PcCDH forms a hydrogen bond to O3 of ABL, and the carbonyl O atom of Ser687 in PcCDH contacts O2 of ABL. The corresponding residues in BcGDHγα are Asn475 and Asn474, respectively, and would be able to recognize glucose as a substrate. In PcCDH, Glu279 and Arg586 recognize the glucose moiety at the reducing end of cellobiose [Glc(β1–4)Glc]. The residue corresponding to Arg586 in PcCDH is Ser365 in BcGDHγα, but a residue corresponding to Glu279 in PcCDH has not been identified in BcGDHγα. Although the most favorable substrate of BcGDHγα is glucose, a large cavity is present in the putative active site of BcGDHγα, as observed in the surface model of BcGDHγα superimposed onto the active site of PcCDH [Supplementary Fig. S6(*d*)], supporting the fact that BcGDHγα recognizes maltose [Glc(α1–4)Glc] as a substrate (Yamashita *et al.*, 2013 ▸).

In contrast, proteins with a similar structure to the γ-subunit were not identified in the *DALI* search. To date, the γ-subunit has been considered to be a hitchhiker protein that promotes the secretion of the catalytic subunit to the periplasm. In the bacterial twin-arginine translocation (TAT) pathway, folded proteins are transported across the bacterial cytoplasmic membrane through the recognition of N-terminal signal peptides containing the twin-arginine motif. In some representative twin-arginine signal peptides, α-helical regions were predicted using the *PSIPRED* secondary-structure prediction method (Palmer *et al.*, 2005 ▸). The γ-subunit of BcGDH contains the twin-arginine motif in the N-terminal signal peptide and was thought to belong to the Tat protein family. Indeed, the structure of the γ-subunit of BcGDH contains five α-helices.

The *DALI* search revealed some proteins with limited homology (Supplementary Table S2 and Fig. S7), including the N-terminal domain (NTD) of *Salmonella typhimurium* chemotaxis receptor methyltransferase (CheR) in complex with *S*-adenosyl-l-homocysteine (SAH), which had the highest *Z*-score (4.7). These domains of the enzymes were reported to be essential for catalytic activity, although they are located far from the catalytic domain and no role as a hitchhiker protein was reported.

The X-ray structure of BcGDHγα also revealed the first structure of a hitchhiker protein in complex with the target protein. According to the results of site-directed mutagenesis studies, the formation of disulfide bonds is required to stabilize the catalytic subunit. In other words, the disulfide bond may prevent denaturation of the 3Fe–4S cluster, thereby maintaining the stability of this enzyme even at temperatures greater than 323 K. Thus, the hitchhiker protein may protect the iron–sulfur cluster until the formation of a complex with the electron-transfer subunit after the catalytic subunit has been secreted into the periplasmic space. The electron-transfer subunit is folded in the periplasmic space and forms a quaternary structure with the catalytic subunit complexed with the hitchhiker protein. However, a mutant catalytic subunit or a mutant hitchhiker protein in the enzyme complex was expressed and functional. These results support the lack of a requirement for disulfide bonds in the functional expression and secretion of the complex into the periplasmic space. Indeed, alignments of the primary structures of the catalytic subunits [Supplementary Fig. S8(*a*)] and hitchhiker proteins [Supplementary Fig. S8(*b*)] of FAD-dependent dehydrogenase complexes reveal that the cysteine residues are not conserved, indicating that the formation of the disulfide bond is not necessary for a complex to form between catalytic subunits and hitchhiker proteins. Therefore, the hitchhiker protein of FAD-dependent dehydrogenase complexes may mainly interact with and recognize the catalytic subunit throughout these interfacial interactions.

Our previous report revealed the presence of a 3Fe–4S cluster in BcGDH (Shiota *et al.*, 2016 ▸). The X-ray structure of the catalytic subunit clearly indicated the position of the 3Fe–4S cluster, which is located on the surface of the catalytic subunit. The distance between N5 of FAD and the 3Fe–4S cluster is about 12–13 Å, which is an adequate distance for electron transfer. These results support our hypothesis that the 3Fe–4S cluster functions in the intramolecular electron transfer from FAD and mediates intermolecular electron transfer from the 3Fe–4S cluster to the electron-transfer subunit. The 3Fe–4S cluster is responsible for electron transfer and interacts with the multi-heme *c* electron-transfer subunit.

As seen in the electron-density map, a covalent bond might form between C8M of FAD and His105 in the α-subunit of BcGDH [Fig. 2 ▸(*b*)]. The position of His105 is conserved in the GMC oxidoreducatase family, such as in P2Oxs (Bannwarth *et al.*, 2004 ▸; Halada *et al.*, 2003 ▸; Hallberg *et al.*, 2004 ▸; Hassan *et al.*, 2013 ▸; Spadiut *et al.*, 2010 ▸; Tan *et al.*, 2013 ▸), COxs (Quaye *et al.*, 2008 ▸) and fumarate reductases B (Iverson *et al.*, 1999 ▸, 2003 ▸; Lancaster *et al.*, 2001 ▸; Madej *et al.*, 2006 ▸), and this residue forms covalent bonds with FAD.

Next, we attempted to predict the position of the electron-transfer subunit by comparing of the structures of CDH and fumarate reductase, considering the previously elucidated intramolecular and intermolecular electron-transfer pathways (Shiota *et al.*, 2016 ▸; Yamashita *et al.*, 2018 ▸), as well as the nature of the electron-transfer subunit (Okuda-Shimazaki *et al.*, 2018 ▸).

In Supplementary Fig. S9, the structure of the catalytic domain of CDH (closed state) was superimposed onto the structure of the α-subunit of BcGDHγα [Supplementary Fig. S9(*d*)]. The 3Fe–4S cluster between the γ- and α-subunits of BcGDH (red dotted circle) is located on the left side, which is the opposite side to the heme *b*-type (magenta stick) electron-transfer domain of CDH. Considering that the primary electron acceptor of FAD is the 3Fe–4S cluster, and that in the next step intermolecular electron transfer occurs between the 3Fe–4S cluster and the electron-transfer subunit, the position of the electron-transfer subunit of BcGDH would be opposite to that of the heme *b*-type electron-transfer domain of CDH in the closed state.

Soluble flavocytochrome *c* fumarate reductase from *Shewanella putrefaciens* is a periplasmic tetraheme flavocytochrome *c* that consists of an N-terminal tetraheme cytochrome *c* domain and a catalytic region that contains the three C-terminal domains. The N-terminal domain containing the tetraheme moiety is connected by an α-helical linker to the FAD-binding catalytic domain with noncovalently bound FAD. Membrane-bound diheme-containing quinol:fumarate reductase (QFR) from *Wolinella succinogenes* is composed of three subunits (A, B and C), in which subunit A contains the catalytic site of fumarate reduction and an FAD covalently bound to His43. Subunit B contains three iron–sulfur clusters and subunit C is a diheme cytochrome *b* (Lancaster *et al.*, 2001 ▸; Madej *et al.*, 2006 ▸).

**Figure 6:**
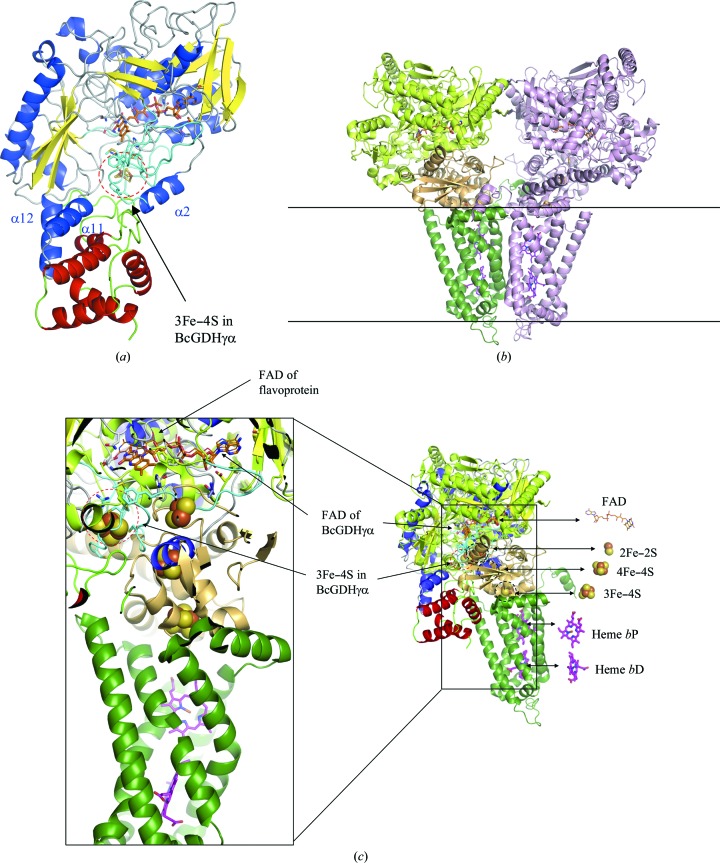
The structures of (*a*) BcGDHγα, (*b*) *W. succinogenes* membrane-bound fumarate reductase (PDB entry 2bs2; Madej *et al.*, 2006 ▸) and the flavoprotein shown in light green in (*b*) were superimposed onto the structure of BcGDHγα in (*c*). The colors of BcGDHγα are the same as in Fig. 2 ▸. The 3Fe–4S cluster is indicated by a red dotted circle. Soluble *S. putrefaciens* fumarate reductase was first superimposed onto the structure of the α-subunit of BcGDHγα with 3.0 r.m.s.d., and the membrane-bound fumarate reductase from *W. succinogenes* was superimposed onto the structure of soluble fumarate reductase from *S. putrefaciens* (with 1.8 r.m.s.d.). Consequently, the structure of the membrane-bound fumarate reductase was directly compared with the structure of BcGDHγα by removing the structure of the soluble fumarate reductase. (*b*) The two-molecule form of fumarate reductase is shown in the crystal structure (Lancaster *et al.*, 1999 ▸). A molecule of fumarate reductase is represented in light pink (right). In the other molecule, subunit A (flavoprotein containing FAD covalently bound to His43), subunit B (iron–sulfur protein including 2Fe–2S, 4Fe–4S and 3Fe–4S clusters) and subunit C (transmembrane-spanning protein including diheme cytochrome *b* molecules) are colored light green, light orange and dark green, respectively. The bound FAD molecules in the structures are shown as orange stick models. The bound FAD and heme *b* molecules are shown as orange lines and magenta stick models, respectively. The iron–sulfur clusters are shown as sphere models. The other molecule of fumarate reductase colored light pink was deleted in (*c*) to clarify the positions of FAD, iron–sulfur clusters and heme *b* molecules, which are related to electron transfer. *b*P is the proximal heme and *b*D is the distal heme.

Focusing on the homology between the soluble flavocytochrome *c* fumarate reductase from *S. putrefaciens* and the membrane-bound fumarate reductase from *W. succinogenes*, as well as the homology between soluble flavocytochrome *c* fumarate reductase from *S. putrefaciens* and the α-subunit of BcGDH, the structure of membrane-bound fumarate reductase from *W. succinogenes* was superimposed onto the structure of the α-subunit of BcGDHγα (Fig. 6 ▸). As shown in the membrane-bound fumarate reductase, the electron is transferred from FAD to the transmembrane protein containing heme *b*P and heme *b*D via 2Fe–2S, 4Fe–S and 3Fe–4S clusters. Interestingly, the positions of FAD and the first Fe–S cluster, 2Fe–2S, of fumarate reductase, with a distance of 12.3 Å, are comparable to those of FAD and the 3Fe–4S cluster of BcGDHγα, with a distance of around 12–13 Å. The distances between Fe–S clusters are 11.0 Å (2Fe–2S and 4Fe–4S) and 9.1 Å (4Fe–4S and 3Fe–4S), whereas the distances from the Fe–S clusters to the heme domains are 17.6 Å (3Fe–4S and cytochrome *b*P) and 15.6 Å (cytochrome *b*P and cytochrome *b*D). Although membrane-bound fumarate reductase contains three Fe–S clusters and two heme domains, the β-subunit of BcGDH contains three heme *c* moieties in its electron-transfer subunit. The intact BcGDH complex including the membrane-bound β-subunit containing three heme *c* moieties may form a similar overall structure to fumarate reductases for effective electron transfer. Interestingly, our previous study on the β-subunit suggested that the electron from the Fe–S cluster is initially transferred to the third heme in the β-subunit (the C-terminal heme domain of the β-subunit), is then transferred to the second heme, is further transferred to the first heme (the N-terminal heme domain of the β-subunit) and is finally transferred to an external artificial electron acceptor. However, when the intact BcGDH is immobilized on the electrode, the electron is transferred from the third heme to the second heme, and is then directly transferred to the electrode. The third heme of BcGDHγαβ may correspond to the position between 4Fe–4S and 3Fe–4S of fumarate reductase, the second heme corresponds to heme *b*P and the first heme corresponds to heme *b*D.

In conclusion, this study reports the first X-ray structure of a representative DET-type FAD-dependent dehydrogenase complex: the BcGDH catalytic subunit complexed with a hitchhiker protein. The structure of BcGDHγα revealed a conserved GMC oxidoreductase-type scaffold and a His/Asn catalytic pair, with a unique structure of the 3Fe–4S cluster, which serves as the electron acceptor of FAD and simultaneously serves as the electron donor for electron transfer of the multiheme *c* subunit. These findings will be essential for improving our understanding of intramolecular and intermolecular electron transfer by DET-type FAD-dependent dehydrogenase complexes, as well as for engineering DET-type enzymes for the development of future bioelectro­chemical devices.

## 5. Related literature   

The following references relate to PDB entries that are mentioned in the supporting information to this article: Batra *et al.* (2016 ▸), Djordjevic & Stock (1997 ▸, 1998 ▸), Golden *et al.* (2014 ▸), Leys *et al.* (1999 ▸), Liu *et al.* (2015 ▸), Mugo *et al.* (2013 ▸), Pitsawong *et al.* (2010 ▸), Salvi *et al.* (2014 ▸), Tan *et al.* (2015 ▸), Wohlfahrt *et al.* (1999 ▸), Yoshida *et al.* (2015 ▸) and Zhang *et al.* (2014 ▸).

## Supplementary Material

PDB reference: γα-subunit complex from *Burkholderia cepacia* FAD glucose dehydrogenase, 6a2u

Supplementary Figures and Table. DOI: 10.1107/S2059798319010878/dw5200sup1.pdf

## Figures and Tables

**Table 1:** Data-collection and refinement statistics Values in parentheses are for the highest resolution bin.

	BcGDHγα	BcGDHγα	SeMet BcGDHγα
Data collection			
Beamline	PF-AR NE3A	PF NW12A	PF-AR NE3A
Temperature (K)	100	100	100
Wavelength (Å)	1.0	1.74086	0.97892
Resolution range (Å)	50.0–2.60 (2.64–2.60)	50.0–2.90 (2.95–2.90)	50.0–3.40 (3.46–3.40)
No. of measured reflections	1273970	369135	929441
No. of unique reflections	60005	43770	49132
Multiplicity	21.2 (21.4)	4.9 (6.8)	18.9 (19.0)
Completeness (%)	99.9 (100.0)	96.7 (99.9)	100.0 (100.0)
Mean *I*/σ(*I*)	24.4 (10.0)	20.2 (5.6)	43.1 (12.6)
*R*_merge_ † (%)	15.0 (42.8)	13.5 (46.9)	16.2 (48.5)
Space group	*P*6_5_22	*P*6_5_22	*P*6_5_22
*a*, *b*, *c* (Å)	110.52, 110.52, 524.88	110.72, 110.72, 525.42	110.48, 110.48, 524.09
α, β, γ (°)	90, 90, 120	90, 90, 120	90, 90, 120
Refinement			
Resolution range (Å)	43.74–2.60 (2.67–2.60)		
No. of reflections	56812 (4078)		
Completeness (%)	99.9 (99.2)		
*R* factor (%)	20.5 (27.3)		
*R*_free_ (%)	26.1 (37.6)		
R.m.s.d., bond lengths (Å)	0.003		
R.m.s.d., bond angles (°)	0.6		
Ramachandran plot			
Most favored region (%)	84.6		
Additional allowed region (%)	15.0		
*B* factors (Å^2^)			
Protein	45.6		
Cofactor FAD	31.6 [two molecules]		
3Fe–4S	33.0 [two molecules]		
Water	29.9		
PDB code	6a2u		

† *R*_merge_ = 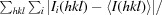 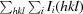, where *I_i_*(*hkl*) is the *i*th measurement and 〈*I*(*hkl*)〉 is the weighted mean of all measurements of *I*(*hkl*).

**Table 2:** Kinetic parameters of BcGDH γα mutants measured at room temperature Plots of the measurements from which the values were calculated are shown in Supplementary Fig. S3.

Sample	*K*_m_ (m*M*)	*V*_max_ (U mg^−1^)
Wild type	0.97 ± 0.052	120 ± 1.5
γα(C213S)	0.76 ± 0.036	76 ± 0.78
γ(C152S)α	0.99 ± 0.067	110 ± 1.8
