# Supplementary material for: X-ray structure of the direct electron transfer-type FAD glucose dehydrogenase catalytic subunit complexed with a hitchhiker protein
Source: Acta Crystallogr D Struct Biol. 2019 Aug 28;75(Pt 9):841–51. doi: 10.1107/S2059798319010878 (PMC6719666; doi:10.1107/S2059798319010878)
Supplement: Supplementary file 1 [file d-75-00841-sup1.pdf]

STRUCTURAL BIOLOGY

Volume 75 (2019)

Supporting information for article:

X-ray structure of the direct electron-transfer-type FAD glucose dehydrogenase catalytic subunit complexed with a hitchhiker protein

Hiromi Yoshida, Katsuhiro Kojima, Masaki Shiota, Keiichi Yoshimatsu, Tomohiko Yamazaki, Stefano Ferri, Wakako Tsugawa, Shigehiro Kamitori and Koji Sode

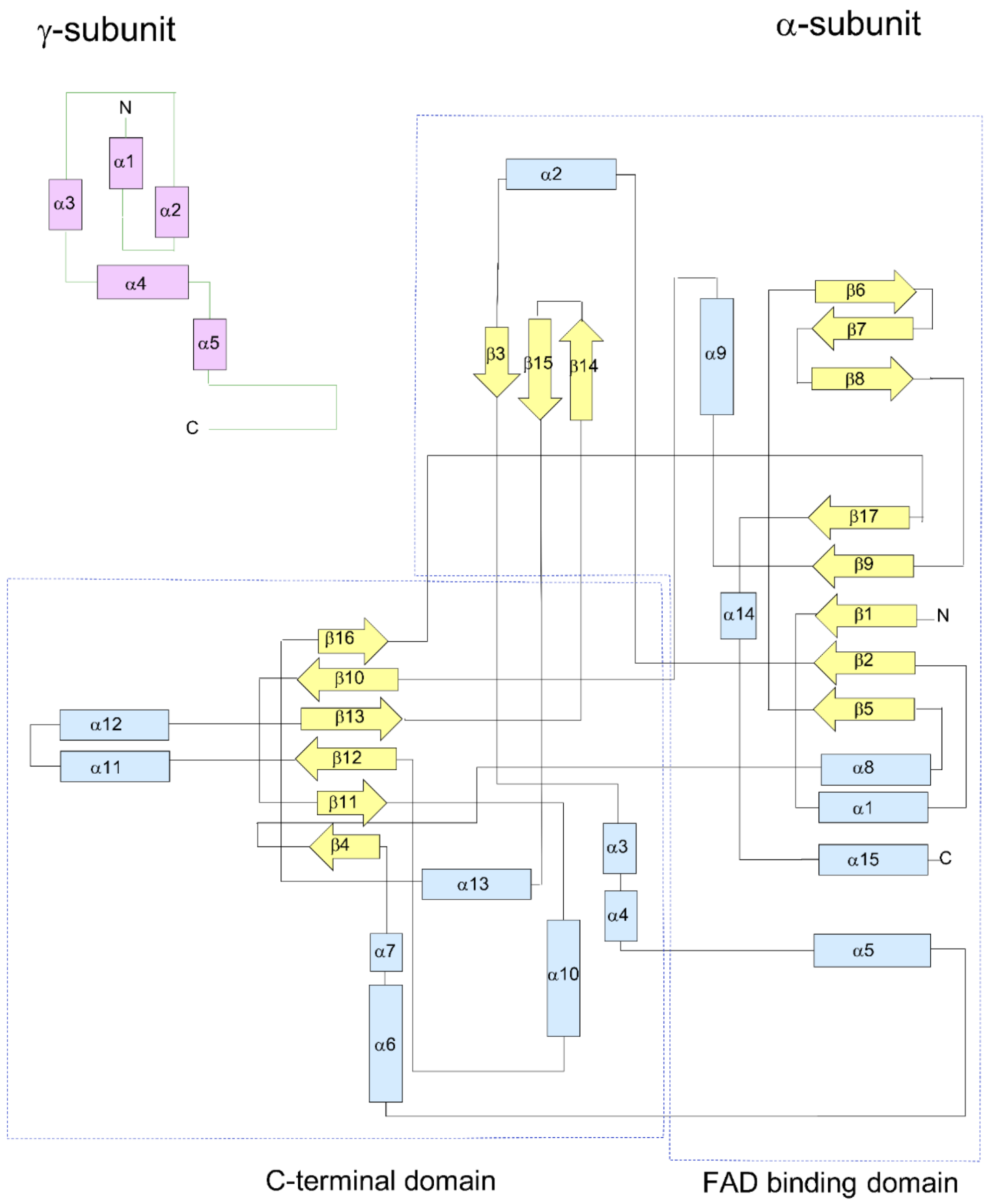

Figure S1 A topological diagram showing the arrangement of secondary structural elements with labels. The β strands and helices are represented by arrows and rectangles, respectively. The α-subunit comprising fifteen α-helices, including two short 3_{10} helices (α7 and α14), and seventeen β-strands adopts an FAD binding fold consisting of three layers ($\beta_3\beta_5\alpha_4$) [(β8, β7, and β6), (β5, β2, β1, β9, and β17), and (α8, α1, α15, and α5)] in a sandwich structure with two α-helices (α9 and α14), and an additional β-sheet (β3, β15, and β14). The additional domain

contains a six-stranded antiparallel β-sheet (β4, β11, β12, β13, β10, and β16) surrounded by six α-helices (α3, α4, α6, α7, α10, and α13) and a loop containing two α-helices (α11 and α12) that protrudes toward the γ-subunit.

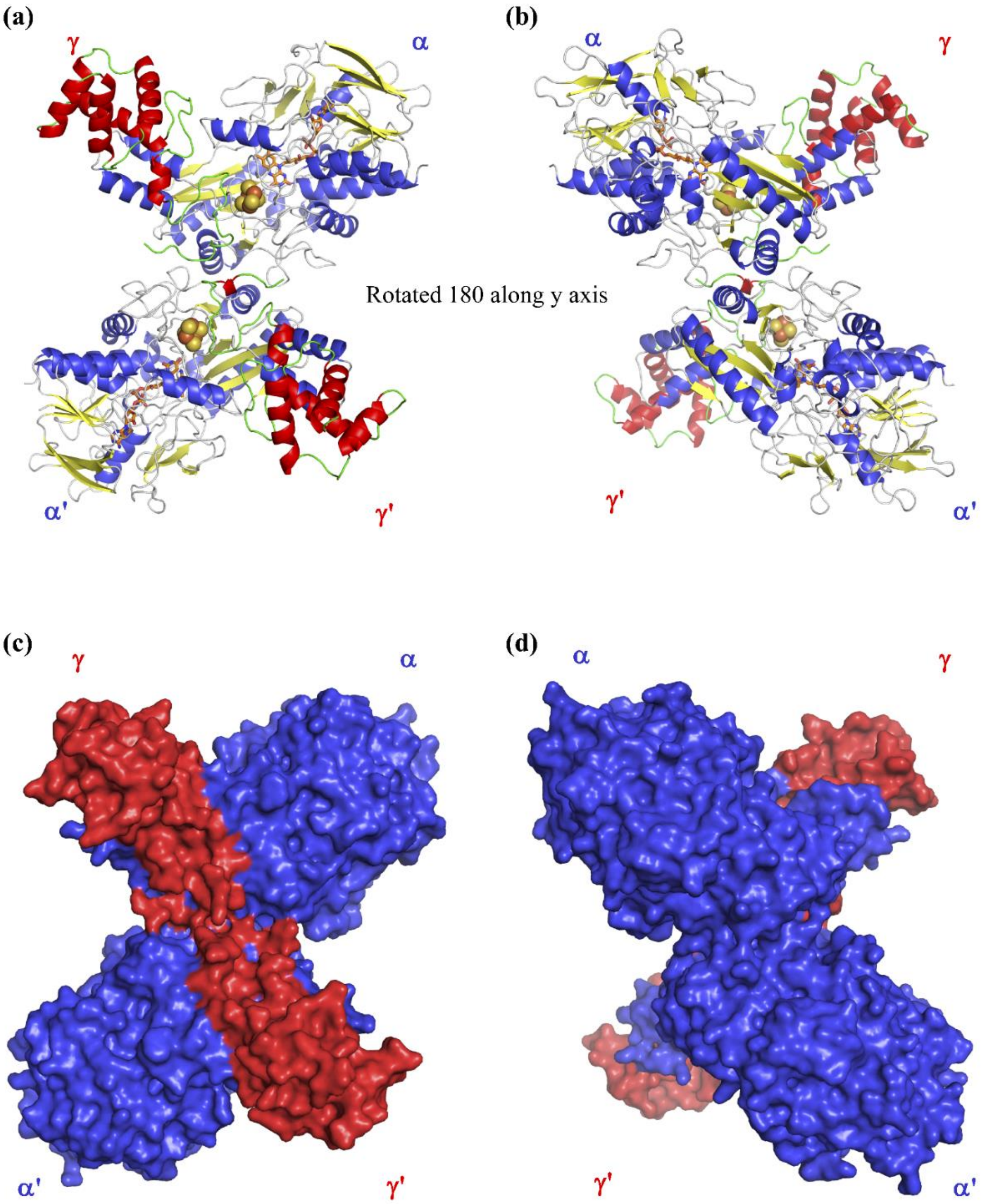

Figure S2 Overall crystal structure of BcGDHγα. Two molecules of BcGDHγα are present in the asymmetric unit. Although the protein appears to form a heterotetramer, the tetrameric structure is unstable and shows two pairs of BcGDHγα according to PISA.

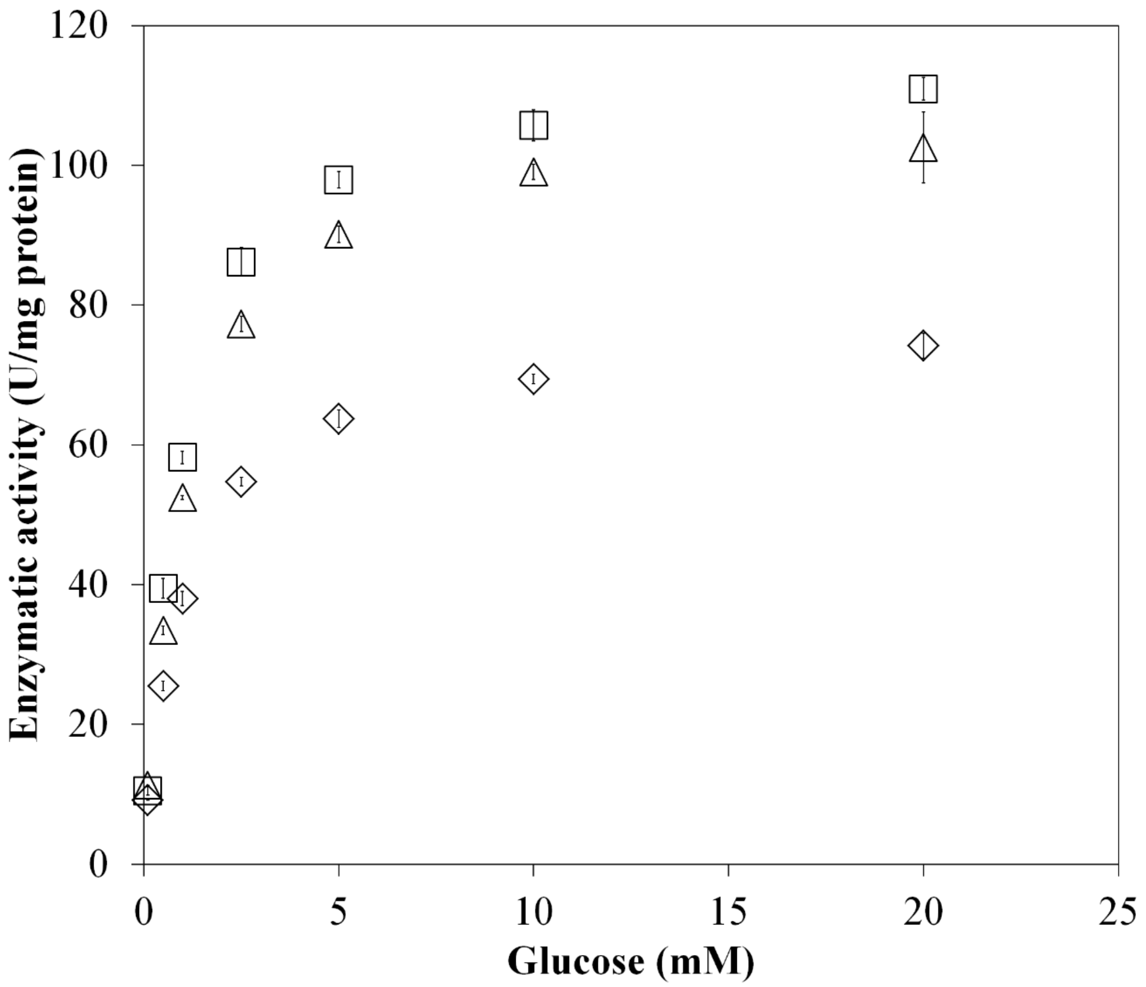

Figure S3 The correlation between the glucose concentration and dye-mediated glucose dehydrogenase activities of wild type (open square), γ(Cys152Ser)α (open triangle) and of γα(Cys213Ser) BcGDHγα (open diamond), respectively.nThe enzyme sample was incubated with 10 mM potassium phosphate buffer (pH 7.0) containing 6 mM 5-methylphenazinium methyl sulfate(phenazine methosulfate (PMS), 0.06 mM 2,6-dichlorophenolindophenol (DCIP), and various concentrations of glucose at room temperature.mThe activity was determined by monitoring the decrease in the absorbance of DCIP at 600 nm and using the molar absorption coefficient of DCIP (16.3 mM cm^{-1} at pH 7.0) to calculate the enzymatic activity.

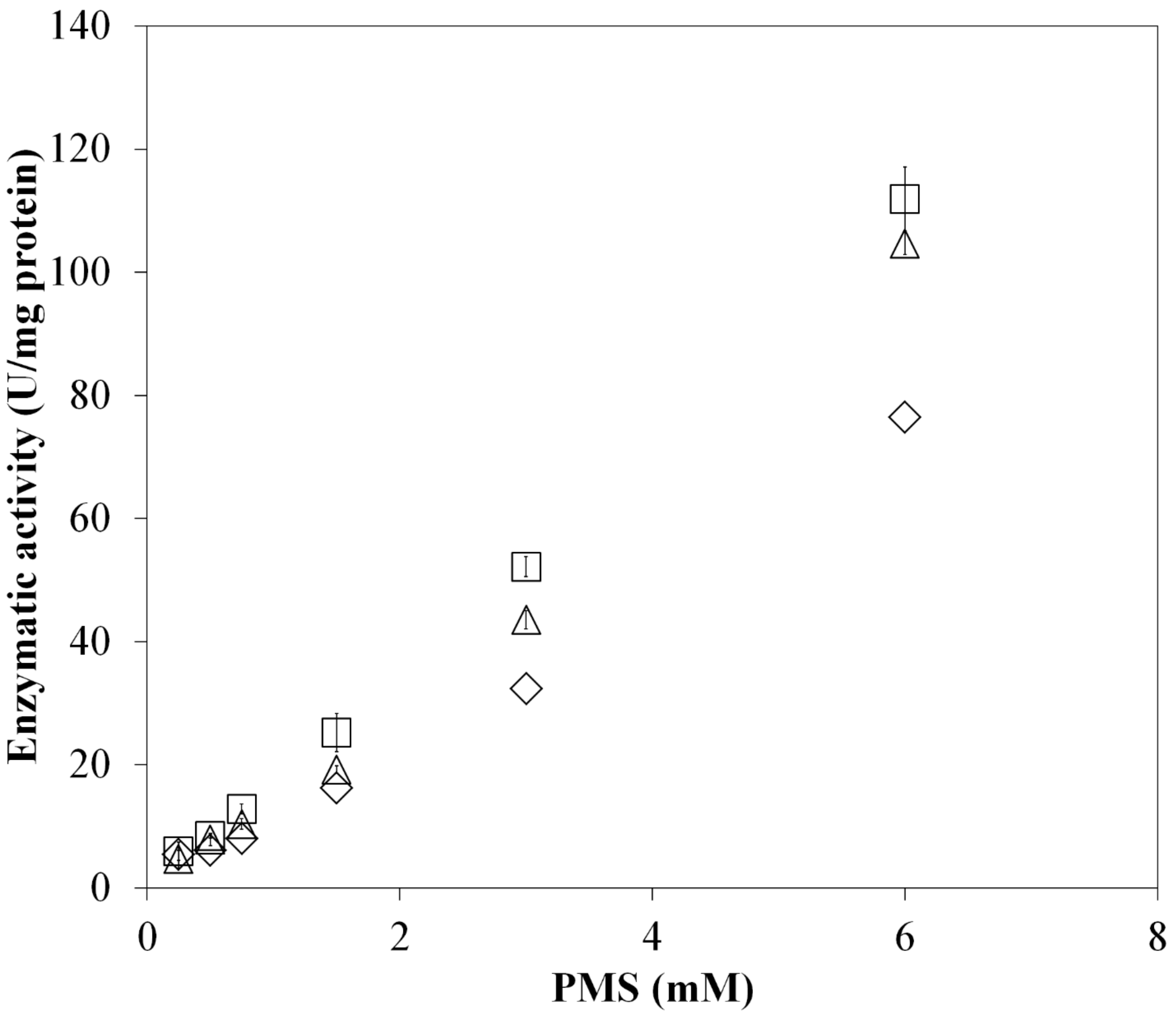

Figure S4 The correlation between the PMS concentration and dye-mediated glucose dehydrogenase activities of wild type (open square), γ(Cys152Ser)α (open triangle) and γα(Cys213Ser) BcGDHγα (open diamond), respectively. The enzyme sample was incubated with 10 mM potassium phosphate buffer (pH 7.0) in the presence of 20 mM glucose, 0.06 mM 2,6-dichlorophenolindophenol (DCIP), and various concentrations of PMS at room temperature. The activity was determined by monitoring the decrease in the absorbance of DCIP at 600 nm and using the molar absorption coefficient of DCIP (16.3 mM cm^{-1} at pH 7.0) to calculate the enzymatic activity.

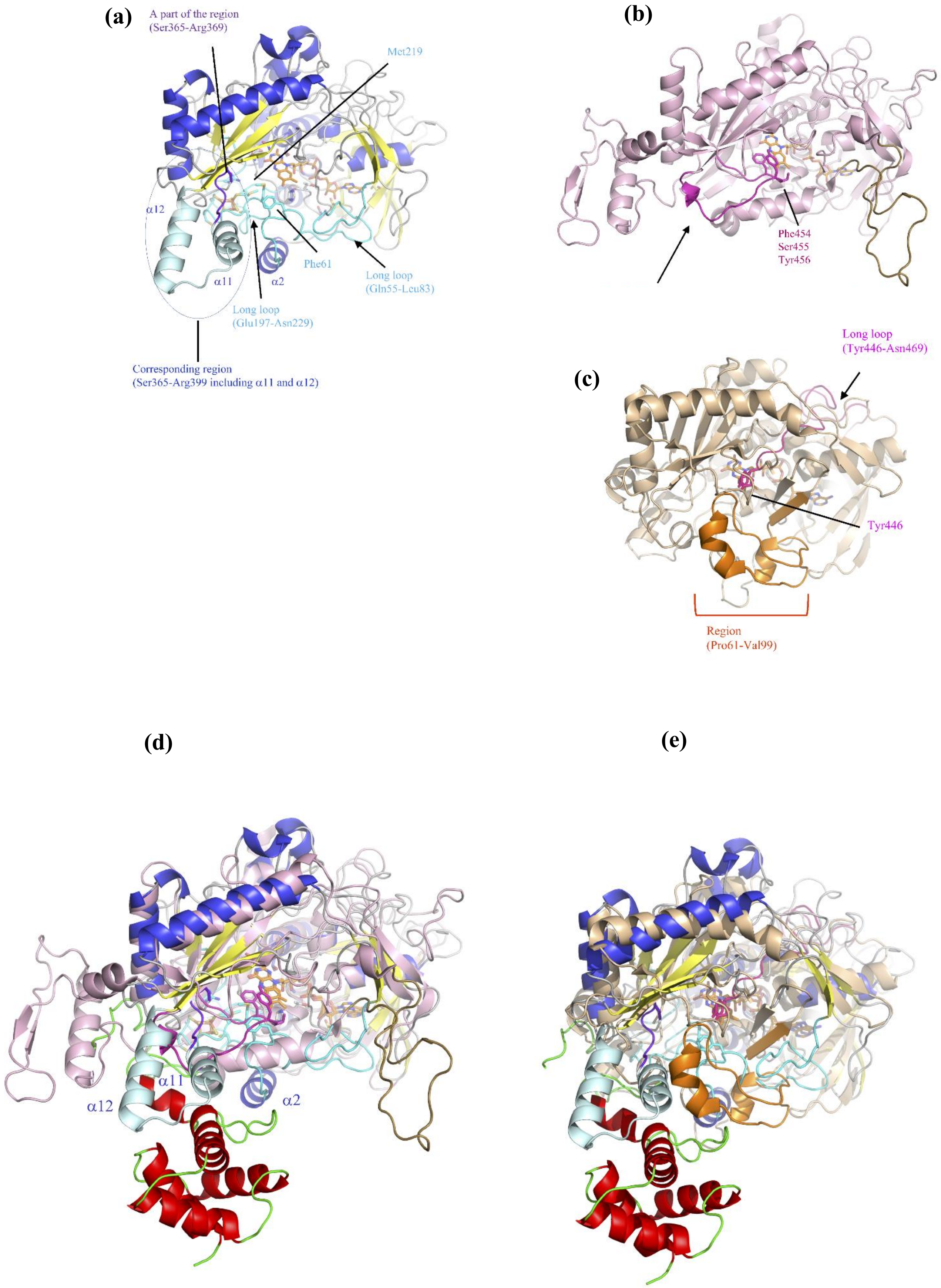
(a)
A part of the region
(Ser365-Arg369)
Met219
α12
α11
α2
Phe61
Long loop
(Gln55-Leu83)
Long loop
(Glu197-Asn229)
Corresponding region
(Ser365-Arg399 including α11 and α12)
(b)
Phe454
Ser455
Tyr456
(c)
Long loop
(Tyr446-Asn469)
Tyr446
Region
(Pro61-Val99)
(d)
α11
α12
α2
(e)

Figure S5 Comparison of the structures of the BcGDH catalytic subunit, pyranose 2-oxidase and cholesterol oxidase. The structures of (a) the α-subunit of BcGDHγα, (b) monomer of homotetrameric pyranose 2-oxidase (P2O) (PDB ID: 3k4c) and (c) cholesterol oxidase (ChOX) (4u2s) are shown. The structures presented in (b) and (c) were superimposed onto the structure of BcGDHγα in (d) and (e), respectively. The colors of BcGDHγα are the same as in Fig. 2, except for the indicated regions. All bound FAD molecules in these structures are shown as orange stick models. In P2O (b), the flexible loop region (Arg451-Leu467) was colored in light magenta, and Phe454-Ser455-Tyr456 residues, which are important for substrate specificity, are shown as a stick model. A long loop region (Val114-Gln146) colored in brown contacts other monomers of P2O and is involved in forming the homotetrameric enzyme. The flexible region of P2O corresponds to the region (Ser365-Arg399) of the BcGDH α-subunit including α11 and α12, which contacts the γ subunit. Two long loop regions (Gln55-Leu83) and (Glu197-Asn229) of BcGDHγα are colored in cyan, including Phe61 (in the former loop) and Met219 (in the latter loop), with a stick model that approaches the isoalloxazine ring of FAD. In ChOX (c), the region corresponding to the latter long loop of the BcGDH α-subunit (Glu197-Asn229) moves away from the isoalloxazine ring and forms short helices and part of the β-sheet (Pro61-Val99, colored in orange). Alternatively, a long loop (Tyr446-Asn469) of ChOX, which is colored in hot pink, approaches the isoalloxazine ring. Tyr446 is represented as a stick model and is located at the *re*-face of the isoalloxazine ring.

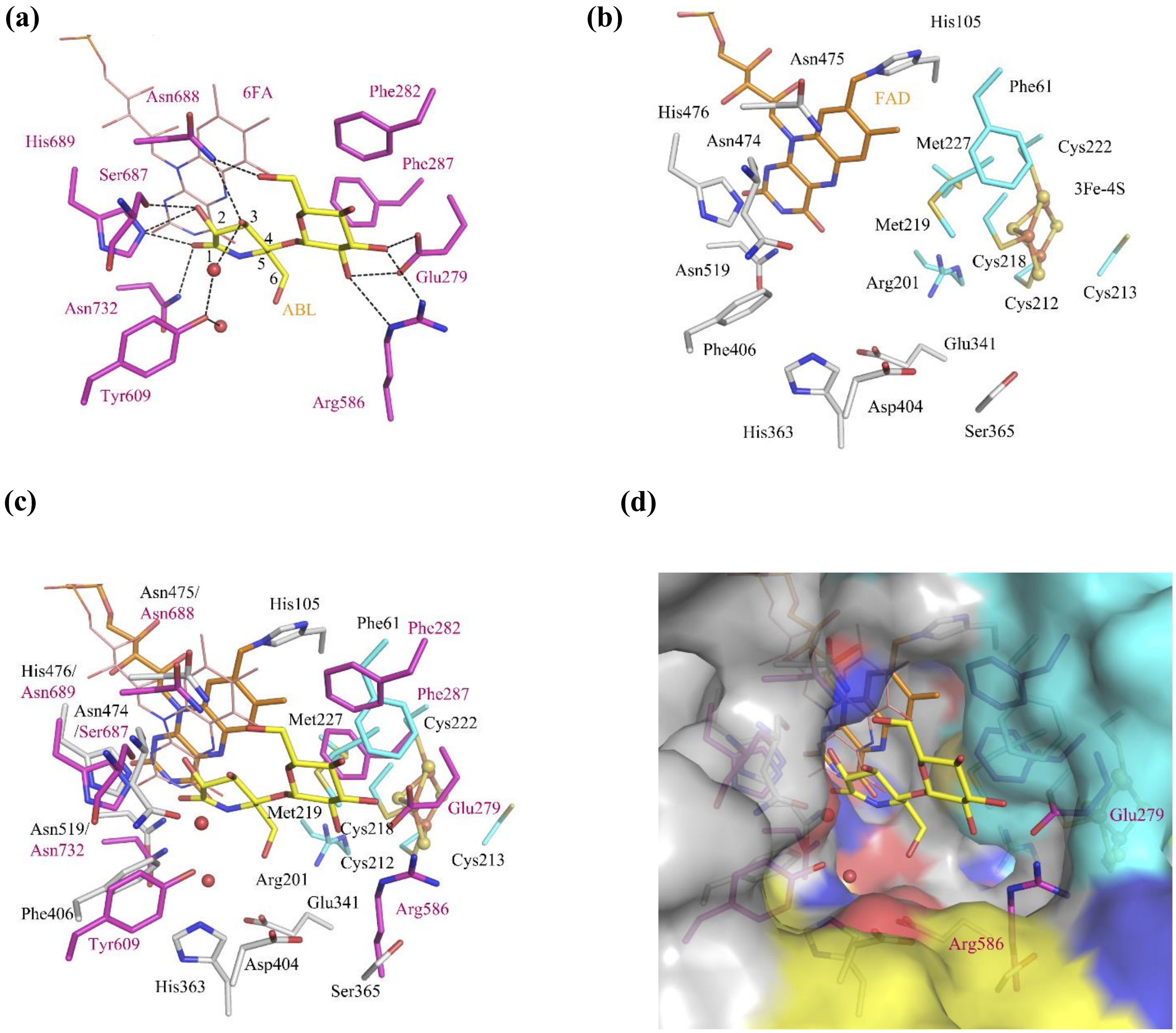

Figure S6 The structure of the putative active site of BcGDHγα. Comparison of the structures of the active sites of (a) the flavoprotein domain of *Phanerochaete chrysosporium* cellobiose dehydrogenase bound to 6-hydroxy FAD (light pink line) and cellobionolactam (inhibitor, ABL, yellow stick) (1naa) (Hallberg et al., J. Biol. Chem., 2003) with the putative active site of (b) BcGDHγα. The residues related to the ligand interactions and the ligand-induced changes are represented as magenta sticks in (a). Water molecules are shown as red spheres. The selected hydrogen bonds are indicated by the dotted lines. The colors of BcGDHγα are the same as in Fig. 2. (c) Comparison of the structures of the active sites of cellobiose dehydrogenase and BcGDHγα. The structure of the active site shown in (a) was superimposed onto the structure shown in (b). (d) Surface model of the BcGDHγα structure shown in (c). A large cavity

is observed in the putative active site of BcGDHγα, suggesting that BcGDHγα recognizes maltose as a substrate.

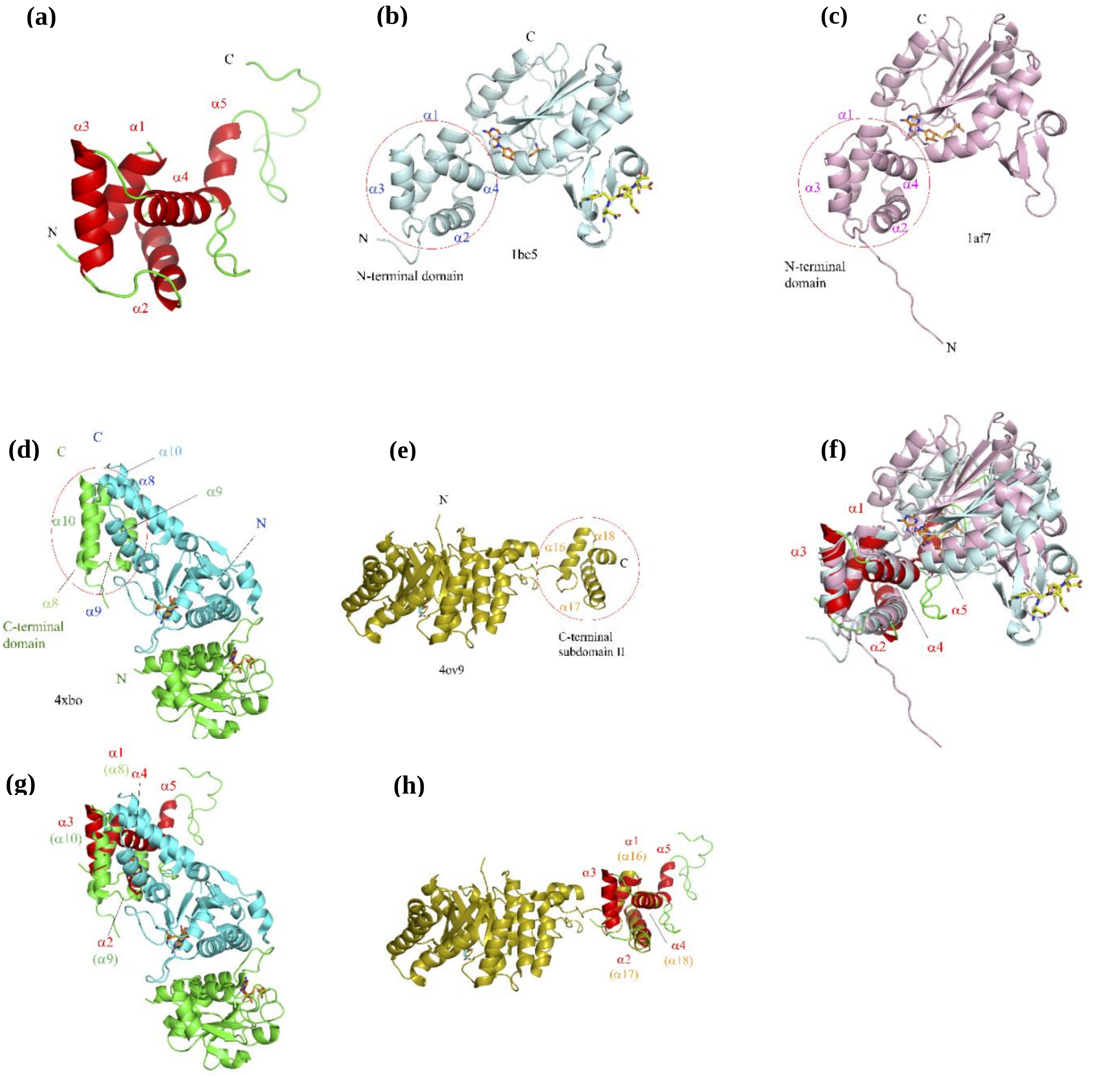

Figure S7 Structures of (a) the γ-subunit of BcGDHγα, (b) *Salmonella typhimurium* N-terminal domain (NTD) of chemotaxis receptor methyltransferase (CheR MTase) with S-adenosyl-L-homocysteine (SAH) and C-terminal pentapeptides of CheR (PDB ID: 1bc5), (c) *S. typhimurium* NTD of CheR MTase with SAH (1af7), (d) *Escherichia coli* C-terminal domain (CTD) of tRNA (cytidine/uridine-2'-O-)-MTase TrmJ with SAH (4xbo), and (e) *Leptospira biflexa* C-terminal subdomain II of isopropylmalate synthase with alpha-isopropylmalate (4ov9). (f) Superimposition of the structures shown in (b) and (c) onto the structure shown in (a). (g) Superimposition of the structure shown in (d) onto the structure shown in (a). (h) Superimposition

of the structure shown in (e) onto the structure shown in (a). In tRNA MTase methyltransferase, two molecules (green and cyan) are shown in (d), since the CTD forms a dimer with other CTDs. The α-helices with numbers indicate the order of α-helices in each molecule. The α-helices corresponding to the γ-subunit of BcGDH are shown. The molecules of SAH are represented as orange sticks in (b, c, d, f, and g). The bound peptides are shown as yellow sticks in (b and f). The bound isopropylmalate is shown as cyan sticks in (e and h).

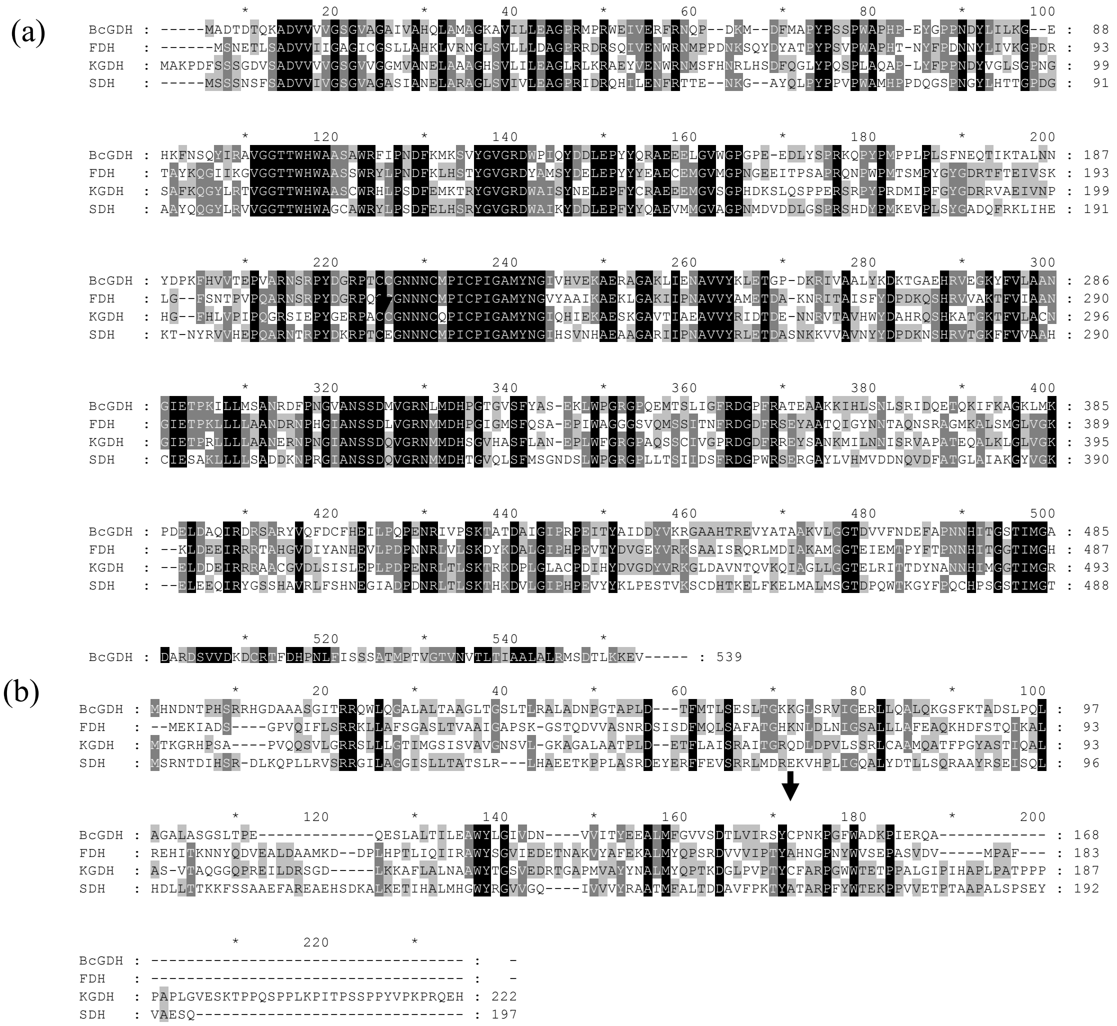

Figure S8 Alignments of catalytic subunits (a) and hitchhiker proteins (b) in FAD oxidoreductases. The Cys residues forming a disulfide bond between the BcGDH catalytic (a) and hitchhiker (g) proteins are indicated with arrows. (a) BcGDH; (GenBank ID: AAN39686.1), FDH; *Gluconobacter japonicus* FDH (GenBank ID: BAM93252.1), KGDH; *G.oxydans* KGDH (GenBank ID: BAM93252.1), SDH; *G.frateurii* sorbitol dehydrogenase (SDH) (GenBank ID: AFW02570.1).(b) BcGDH; (GenBank ID: CAZ78686.1/WP_006396897.1), FDH; *G.japonicus* FDH (GenBank ID: BAM93250.1), KGDH; *G.oxydans* KGDH (GenBank ID: BAQ21462.1), SDH; *G.frateurii* sorbitol dehydrogenase (SDH) (GenBank ID: BAD60912.1).

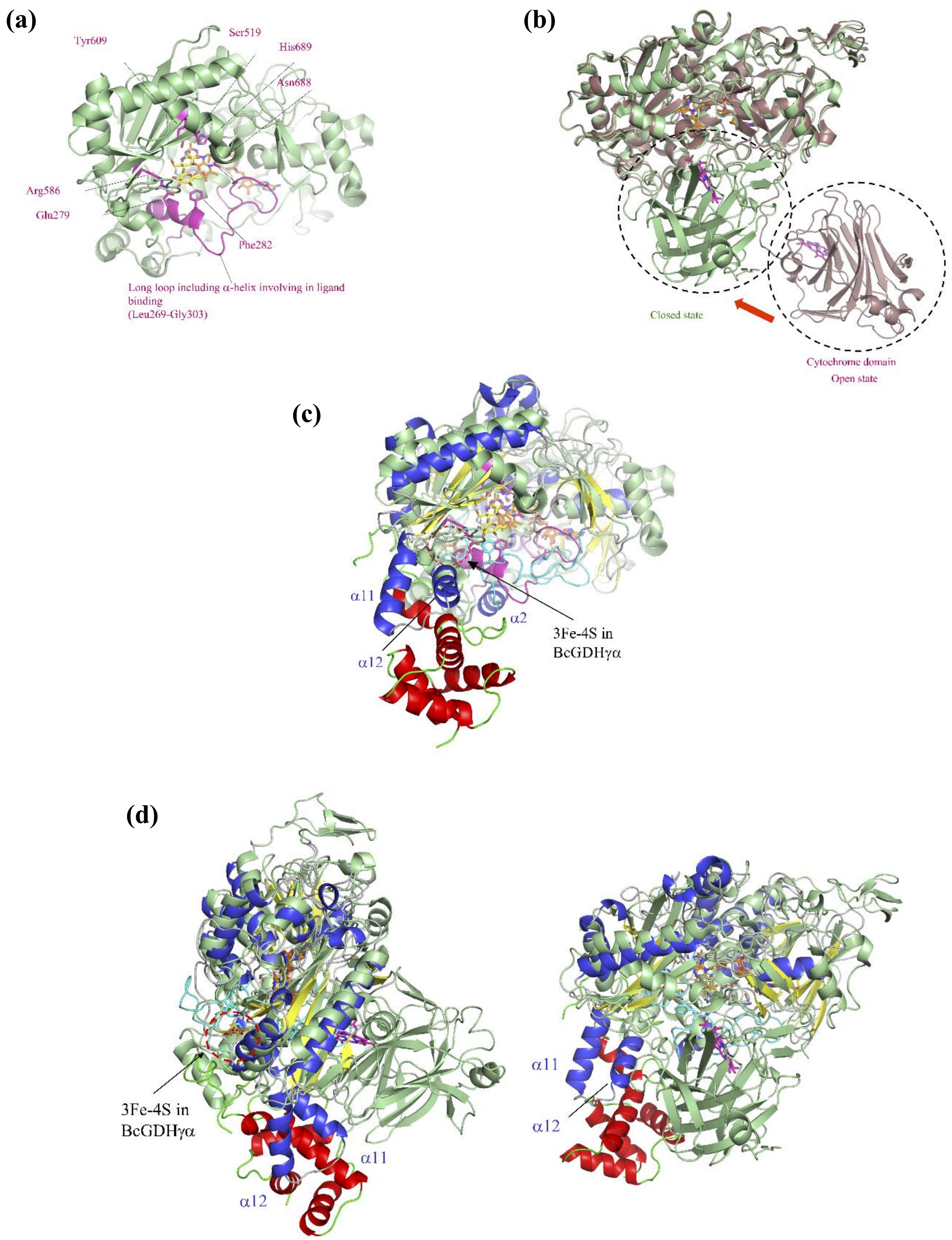
(a)
Tyr609
Ser519
His689
Asn688
Arg586
Glu279
Phe282
Long loop including α-helix involving in ligand binding
(Leu269-Gly303)
(b)
Closed state
Cytochrome domain
Open state
(c)
α11
α2
α12
3Fe-4S in
BcGDHγα
(d)
3Fe-4S in
BcGDHγα
α11
α12
α11
α12

Figure S9 The structures of (a) the flavoprotein domain of *Phanerochaete chrysosporium* cellobiose dehydrogenase bound to 6-hydroxy FAD (orange sticks) and cellobionolactam (inhibitor, yellow sticks) (1naa), (b) *Myricoccum thermophilum* cellobiose dehydrogenase with a heme b-binding cytochrome domain (4qi6, closed state, pale green) and *Neurospora crassa* cellobiose dehydrogenase with a cytochrome domain (4qi7, open state, pale violet). The residues related to the ligand interactions and the ligand-induced changes are represented in magenta sticks in (a). The bound FAD and cytochrome b are shown in orange and magenta sticks, respectively. Cytochrome b domains are indicated by black dotted circles. The structure shown in (a) and the closed state of the structure shown in (b) were superimposed onto the structure of BcGDHγα in (c) and (d), respectively. The colors of BcGDHγα are the same as in Fig. 2. The FAD molecules are shown as orange stick models in the structures.

Table S1 Structural comparisons of α-subunit of BcGDH with the Glucose-Methanol-Choline oxidoreductase (GMC) family according to the Dali search.

Each representative organism and PDB ID in each protein family is shown with the highest Z-score, RMSD and amino acid sequence identity. The values of scores in parentheses are summarized as found as belonging to the same protein family in Dali search.

Structurally similar proteins<br>Reported oligomer state	Representative organism<br>(PDB ID)	Z-score	r.m.s.d.	% id
Pyranose 2-oxidases<br>Homo tetramer	*Trametes ochracea*<br>(3k4c)	33.0<br>(28.6~33.0)	2.6<br>(2.6~2.7)	18<br>(18~19)
Cholesterol oxidases<br>Monomer	*Streptomyces* sp.<br>(4u2l)	31.0<br>(30.1~31.0)	2.8<br>(2.7~2.9)	15<br>(14~16)
Pyridoxine 4-oxidases<br>Homo dimer	*Mesorhizobium loti*<br>(3t37)	30.1<br>(30.1)	2.8<br>(2.8)	19<br>(16~19)
Choline oxidases<br>Monomer	*Arthrobacter globiformis*<br>(4mjw)	29.5<br>(29.1~29.5)	2.8<br>(2.6~2.8)	19<br>(19~20)
Cellobiose dehydrogenases<br>Monomer	*Phanerochaete chrysosporium*<br>(1naa)	28.8<br>(26.0~28.8)	3.2<br>(3.2)	18<br>(18~19)
5-hydroxymethylfurfural oxidases<br>Monomer	*Methylovorus* sp.<br>(4udp)	28.7<br>(28.0~28.7)	2.8<br>(2.7~2.8)	18<br>(18)
Hydroxynitrile lyases<br>Monomer	*Prunus dulcis*<br>(5eb4)	27.4<br>(26.7~27.4)	3.0<br>(2.9~3.4)	16<br>(16~17)
Aryl alcohol oxidases<br>Monomer	*Pleurotus eryngii*<br>(3fim)	27.2<br>(27.2)	2.9<br>(2.9)	16<br>(16)
Mimivirus R135<br>Homo dimer	*Acanthamoeba polyphaga* mimivirus<br>(4z26)	27.0<br>(23.6~27.0)	3.4<br>(3.3~3.4)	18<br>(18)
Glucose dehydrogenases<br>Monomer	*Aspergillus flavus*<br>(4ynu)	26.8<br>(26.4~26.8)	2.6<br>(2.6)	18<br>(18~19)
Glucose oxidases<br>Homo dimer	*Penicillium amagasakiense*<br>(1gpe)	25.5<br>(25.2~25.5)	2.8<br>(2.8)	16<br>(16~17)
Methanol oxidases<br>Homo octamer	*Pichia pastoris*<br>(5hsa)	23.8<br>(23.4~23.8)	3.0<br>(3.0)	15<br>(15)
Flavocytochrome C fumarate reductases<br>Monomer	*Shewanella putrefaciens*<br>(1d4d)	18.1<br>(17.0~18.1)	3.1<br>(3.1)	18<br>(18~19)

Table S2 Structural comparisons of γ-subunit of BcGDH according to the Dali search.

Each representative organism and PDB ID in each protein family is shown with the highest Z-score, RMSD and amino acid sequence identity.

Structurally similar proteins	Representative organism (PDB ID)	Z-score	r.m.s.d.	% id
N-terminal domain (NTD) of chemotaxis receptor methyltransferase (CheR) with S-adenosyl-L-homocysteine (SAH) and C-terminal pentapeptides of the chemotaxis receptors	*Salmonella typhimurium* (1bc5)	4.7	5.3	7
NTD of CheR with SAH	*Salmonella typhimurium* (1af7)	4.6	2.6	12
C-terminal domain (CTD) of tRNA (cytidine/uridine-2'-O-)-methyltransferase TrmJ with SAH	*Escherichia coli* (4xbo)	4.2	3.0	4
C-terminal subdomain II of isopropylmalate synthase with alpha-isopropylmalate	*Leptospira biflexa* (4ov9)	4.1	2.7	11
NTD of glutamate O-methyltransferase in complex with SAH	*Bacillus subtilis* (5ftw)	4.0	3.1	24
RNA polymerase sigma-H factor	*Fusobacterium nucleatum* (3mzy)	3.9	3.1	11
